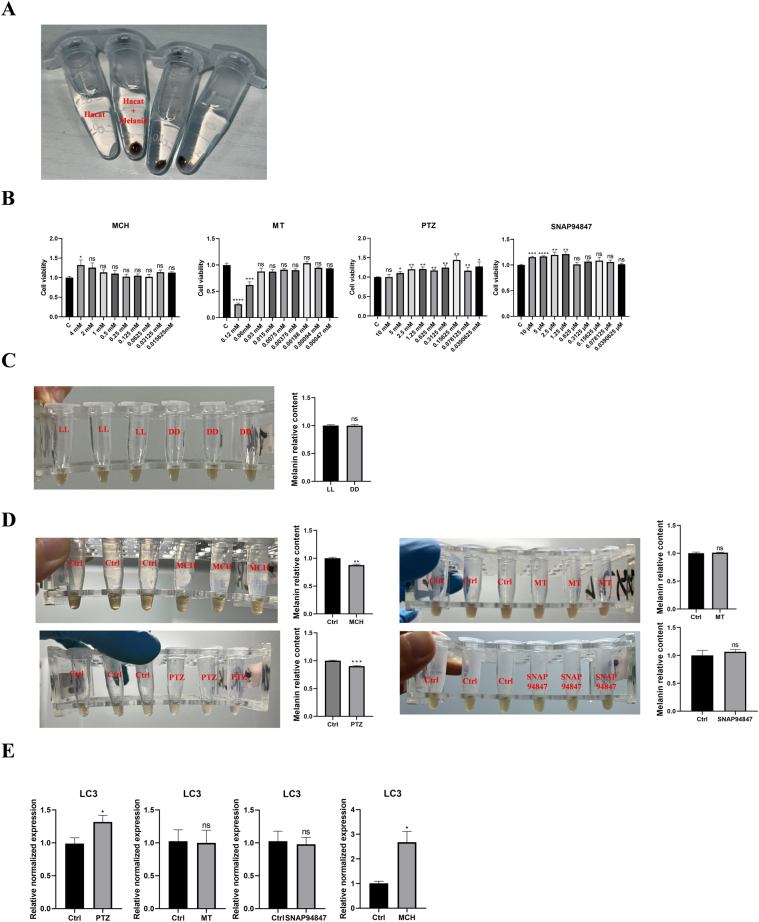# Melanin concentrating hormone-sleep pressure loop regulates melanin degradation through both autophagic degradation and lysosomal hydrolysis in zebrafish

**DOI:** 10.1016/j.jbc.2025.108486

**Published:** 2025-04-08

**Authors:** Qingquan Guo, Yudong Zhang, Jianhua Zhang, Xiaoyu Tian, Yawen Zhou, Yaxin Wang, Mingjie He, Lu Chen, Jiaqi Zeng, Chuanjin Tang, Qiuru Li, Zhenming He, Bingji Ma, Chenyang Jiang, Haishan Zhao

**Affiliations:** 1 School of Chemical Engineering and Light Industry, Guangdong University of Technology, Guangzhou, Guangdong, China; 2 N.O.D Topia (GuangZhou) Biotechnology Co., Ltd, Guangzhou, Guangdong, China; 3 Joint Laboratory for Translational Cancer Research of Chinese Medicine of the Ministry of Education of the People's Republic of China, Guangdong Key Laboratory for translational Cancer research of Chinese Medicine, International Institute for Translational Chinese Medicine, School of Pharmaceutical Sciences, Guangzhou University of Chinese Medicine, Guangzhou, Guangdong, China; 4 Department of Allergy and Clinical Immunology, National Clinical Research Center for Respiratory Disease, State Key Laboratory of Respiratory Disease, Guangzhou Institute of Respiratory Health, the First Affiliated Hospital of Guangzhou Medical University, Guangzhou Medical University, Guangzhou, Guangdong, China; 5 Guangzhou Sun-Hi Biotechnology Co., Ltd, Guangzhou, Guangdong, China; 6 Medical Research Institute, Guangdong Provincial Key Laboratory of South China Structural Heart Disease, Guangdong Provincial People’s Hospital (Guangdong Academy of Medical Sciences), Southern Medical University, Guangzhou, Guangdong, P.R. China

**Keywords:** melanin-concentrating hormone, sleep pressure, whitening, zebrafish, degradation

## Abstract

Melanin-concentrating hormone (MCH) is a cyclic peptide initially isolated from salmon and later found to be conserved in mammals. It plays a role in regulating melanin changes and rhythmic behaviors such as sleep and feeding, though its relationship with these processes is not fully understood. Our preliminary research revealed significant differences in melanin degradation in zebrafish under varying light conditions, suggesting a link to MCH. This study aims to explore MCH's role in lighting-induced changes in rhythmic behavior patterns and melanin of zebrafish. Using the zebrafish model, we evaluated MCH expression under different lighting conditions and analyzed the effects of arousal-promoting and sleep-inducing agents. We also investigated the impact of exogenous MCH and its inhibitors on melanin degradation, behavioral changes, and differences in MCH expression to uncover potential regulatory relationships between MCH, sleep pressure, and melanin. In-depth research using flow cytometry, acridine orange staining, LysoTracker Red staining, and quantitative real-time PCR analysis of autophagy- and apoptosis-related genes showed that melanin degradation regulation depends on MCH expression levels. Sleep pressure can intervene in MCH's effects, forming a regulatory loop to jointly regulate melanin degradation. The influence of the MCH-sleep pressure loop on melanin degradation is closely tied to autophagic and lysosomal pathways. Our findings reveal a mutually regulatory loop in zebrafish between MCH and sleep pressure, affecting melanin degradation through these pathways.

Melanin-concentrating hormone (MCH) is a cyclic peptide, consisting of 17 amino acids in bony fish, with its origins dating back to the 1950s when it was first discovered and isolated from salmon. It was postulated that teleost fish possess a dual rapid mechanism for skin adaptation to environmental backgrounds, with MCH responsible for melanin aggregation to lighten skin color and melanocyte-stimulating hormone (MSH) responsible for melanin dispersion to darken skin color (1, 2). When teleost fish are in bright or light-colored backgrounds, hypothalamic neurons secrete MCH, which projects axons to the neurohypophysis (3), leading to elevated plasma MCH concentrations. MCH acts on melanophores, aggregating pigment-containing melanosomes around the nucleus, thereby altering the refractive index of fish scales and lightening skin color (4). This indicates that MCH can be released as a true neurohypophysial hormone into the circulation, acting on dermal melanocytes (5). Six years after the primary structure of salmon MCH was elucidated, its homolog, a 19-amino acid cyclic peptide, was characterized in rats by Vaughan’s group (6), marking the discovery of MCH in mammals. Regarding research on MCH and melanin in mammals, studies using the B16 mouse melanoma cell model have suggested that MCH may not act directly on melanocytes but may inhibit melanin production by activating the MCH-Agouti-related pathway (7). Currently, research on MCH neurons in mammals also involves areas such as sleep-wake cycles, feeding behavior, and energy balance.

The MCH neuronal system is considered a sleep-promoting system active during the day, leading to higher MCH levels in the cerebrospinal fluid, and inactive after falling asleep (8, 9). Studies have shown that injecting MCH into the cerebral ventricles increases rapid eye movement sleep (REM sleep) and slow-wave sleep (nonrapid eye movement, NREM sleep) in a dose-dependent manner (10). Similarly, treatment with MCH receptor R1 antagonists decreases REM sleep and NREM sleep (11). However, how MCH affects melanin and the relationship between sleep pressure and melanin has not been clearly reported.

The early embryos of zebrafish are transparent, allowing for the observation of melanin within the body without complex experimental procedures. Due to the essentially identical melanin-related processes in the embryos compared to those in humans, many researchers use zebrafish for phenotypic screening to discover compounds that regulate melanin production, making zebrafish a common model for evaluating the effectiveness of whitening treatments (12). During the developmental stage of 3 to 5 days postfertilization (dpf), rhythmic patterns gradually form in zebrafish embryos, and most genes related to rhythm are conserved in zebrafish (13), making this model also suitable for studies on rhythm and sleep.

In the preliminary exploration of this study, we randomly assigned embryos to constant light (LL), constant darkness (DD), and normal light-dark cycle (LD) environments and observed the surface melanin after the treatment. The results showed that compared to the LD group, the surface melanin of embryos in the LL group significantly decreased, while in the DD group it significantly increased. We further repeated the experiment using Holt-Buffer containing the melanin synthesis inhibitor 1-phenyl 2-thiourea (PTU), and the results remained the same. Quantitative analysis indicated that melanin content significantly decreased in the LL group, slightly decreased but not significantly in the LD group, and significantly increased in the DD group. Combining the research on MCH in bony fish, we speculate that the different levels of melanin degradation under various lighting environments may be related to MCH. Reviewing the research on MCH in the field of sleep-wakefulness, LL, LD, and DD environments typically correspond to sleep deprivation, normal sleep, and excessive sleep environments in sleep research, respectively, leading us to suspect the role of sleep pressure and MCH in the regulation of melanin degradation (14).

Therefore, this study will employ the zebrafish model to investigate the following aspects: (1) the impact of MCH on melanin degradation; (2) the impact of sleep pressure on melanin degradation; (3) the interaction between MCH and sleep pressure in the regulation of melanin degradation; (4) the specific mechanisms by which MCH and sleep pressure affect melanin degradation.

## Results

### Different light levels affect the level of melanin degradation

**Figure 1:**
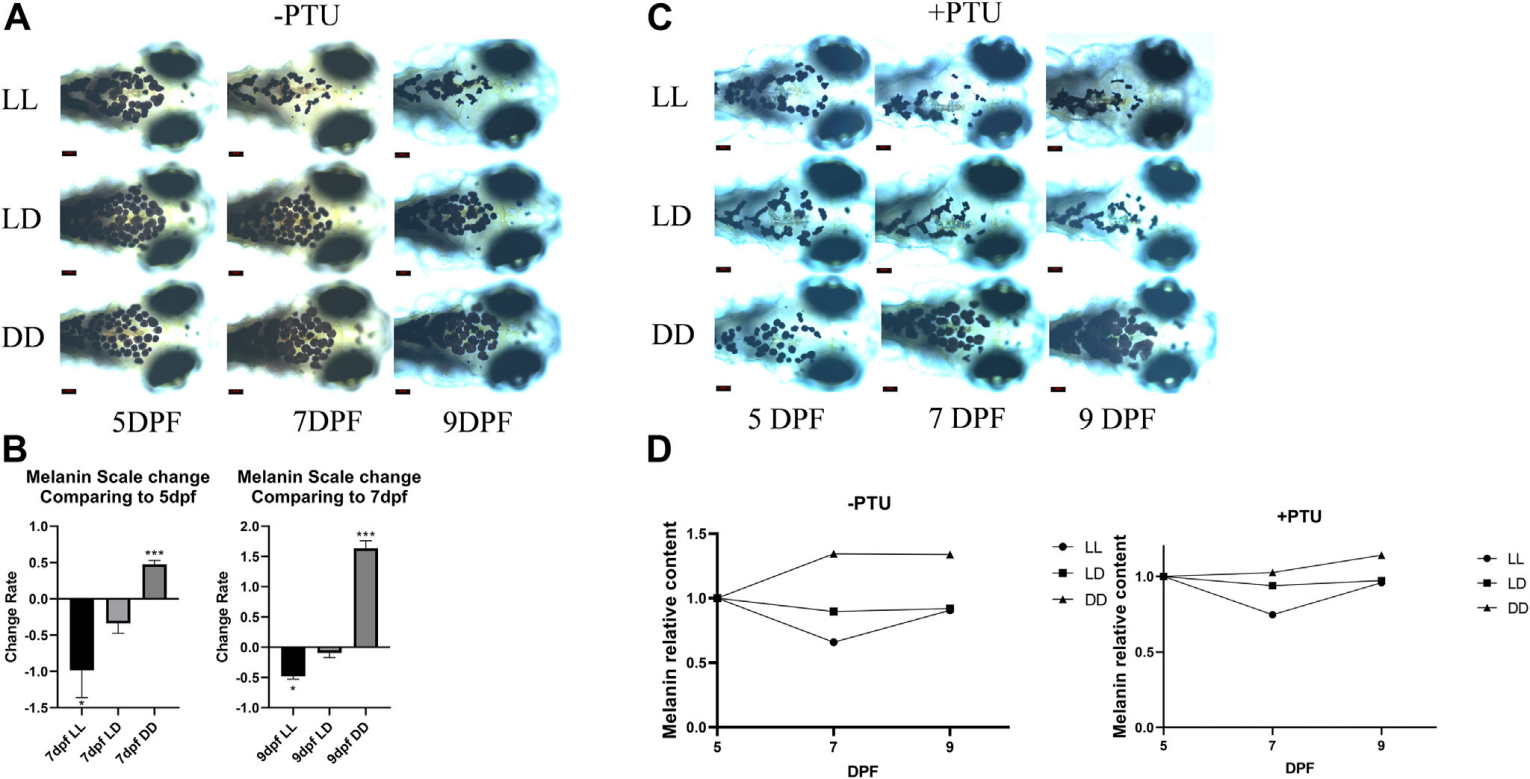
**The impact of different lighting conditions on the degradation of zebrafish melanin.***A*, surface melanin of LL, LD, and DD at 5, 7, and 9 dpf without PTU. *B*, scale changes in surface melanin area of LL, LD, and DD at 7 dpf compared to 5 dpf and 9 dpf compared to 7 dpf. *C*, surface melanin of LL, LD, and DD at 5, 7, and 9 dpf with PTU. *D*, melanin content of LL, LD, and DD at 5, 7, and 9 dpf. Surface melanin area was recorded for the same embryo at 5, 7, and 9 dpf, data are expressed as mean ± SEM (n = 8). ∗*p* < 0.05, ∗∗∗*p* < 0.001, *versus* 7 dpf LD or 9 dpf LD. dpf, days postfertilization; LD, light-dark cycle, PTU, 1-phenyl 2-thiourea.

Our preliminary experimental results indicate that zebrafish embryos under LL conditions exhibit a significant reduction in melanin area on their body surface. Under LD conditions, the melanin area also decreases, but to a lesser extent than under to LL conditions. In contrast, under DD conditions, the melanin area shows an increasing trend (Fig. 1, *A* and *B*). The addition of the classic melanin synthesis inhibitor PTU to Holt-Buffer resulted in melanin area changes consistent with those observed in the control group without PTU (Fig. 1*C*). Quantitative analysis of melanin content in LL, LD, and DD environments at 5, 7, and 9 dpf revealed results consistent with the phenotypic changes in melanin area. Compared to the melanin content at 5 dpf, there was a significant decrease in melanin content under LL conditions, no significant change under LD conditions, and a significant increase under DD conditions (Fig. 1*D*). These results confirm that changes in melanin area are consistent with changes in content. Therefore, this study preliminarily concludes that different light levels affect the level of melanin degradation.

Meanwhile, according to the experimental results, the level of melanin change at 7 dpf is more significant than at 9 dpf. Therefore, this study selects 7 dpf as the end point for subsequent experiments.

### MCH regulates the level of melanin degradation

**Figure 2:**
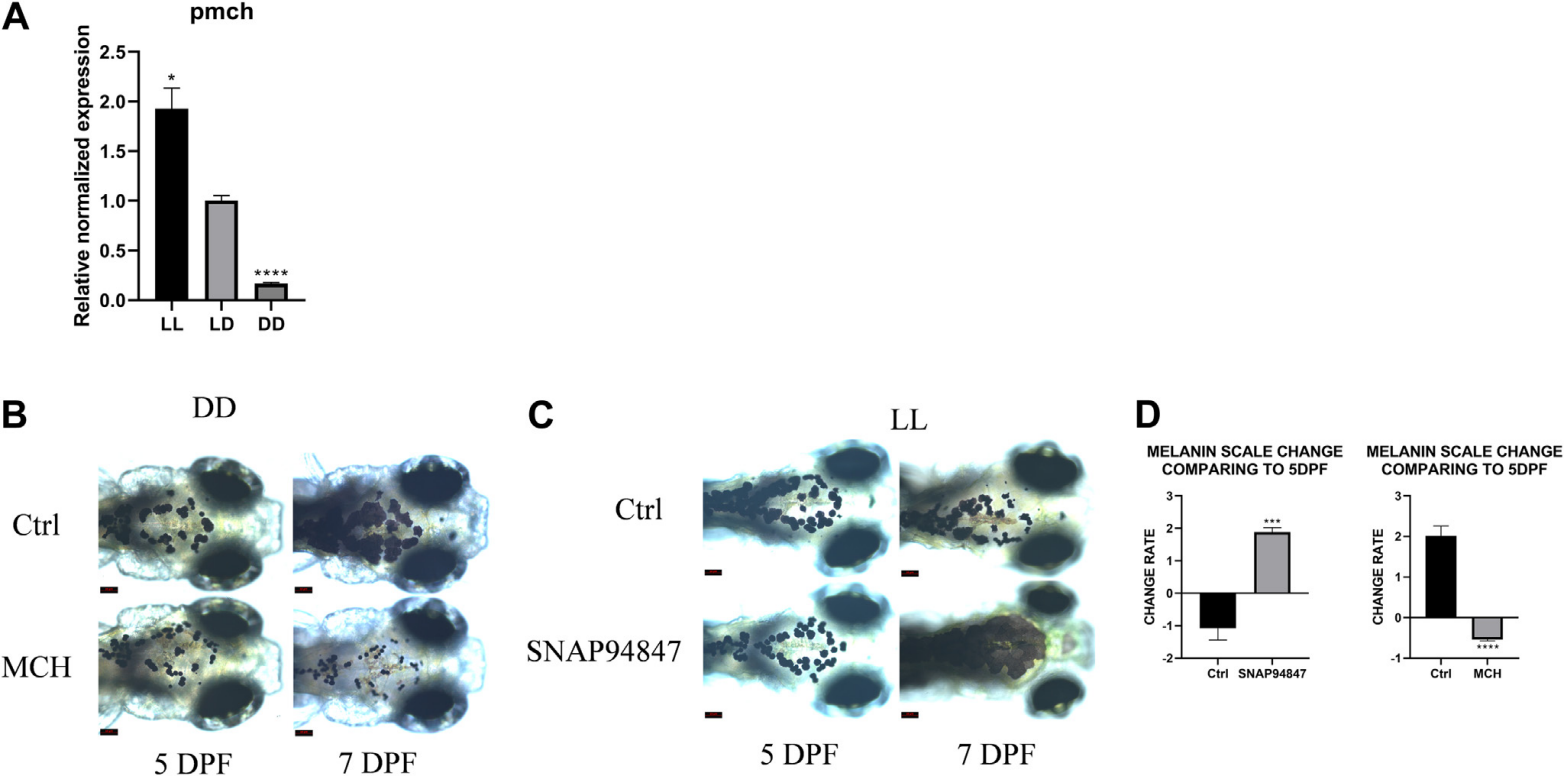
**Light patterns alter the secretion of MCH, thereby affecting the degradation of melanin**. *A*, relative normalized expression of pmch in LL, LD, and DD at 7 dpf. *B*, surface melanin of Ctrl and MCH groups at 5 dpf and 7 dpf under DD. *C*, surface melanin of Ctrl and SNAP94847 groups at 5 dpf and 7 dpf under LL. *D*, scale changes in surface melanin area of SNAP94847 group, MCH group, and Ctrl group at 7 dpf compared to 5 dpf. Data are expressed as mean ± SEM (n = 6). ∗∗∗*p* < 0.001, ∗∗∗∗*p* < 0.0001, *versus* LD or Ctrl. dpf, days postfertilization; LD, light-dark cycle, MCH, melanin-concentrating hormone.

**Fig. S1:**
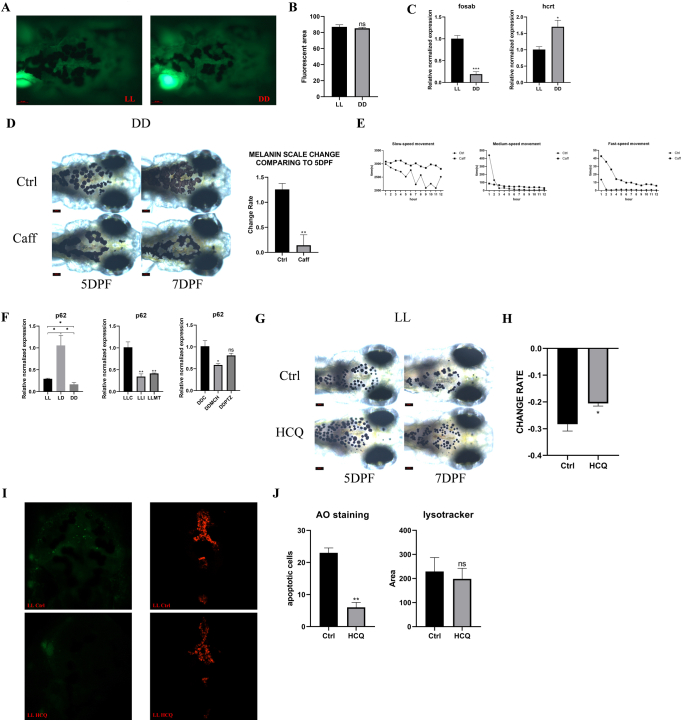


Through a literature review, this study found that the differences in melanin degradation under different light conditions (LL, LD, and DD) are closely related to a peptide substance known as MCH. When the embryo perceives light signals, its hypothalamus secretes MCH, which promotes the aggregation of melanin spots. Using quantitative real-time PCR (RT-qPCR), we detected the expression levels of the precursor gene pmch of MCH peptide under LL, LD, and DD conditions and found that the pmch expression levels showed a gradually decreasing trend among the three groups, with statistically significant differences between groups (Fig. 2*A*). We also detected the levels of oxidative stress under LL and DD conditions using the 2',7'-dichlorofluorescein diacetate (DCFH-DA) probe and found no significant differences (Fig. S1, *A*–*B*). In further experiments, we set up a control group and an MCH peptide group under DD conditions, and a control group and a group treated with the MCH inhibitor SNAP94847 under LL conditions. Comparing the changes in melanin area at 7 dpf and 5 dpf, we found that under DD conditions, the MCH group had a significantly reduced melanin area compared to the control group; under LL conditions, the SNAP94847 group had a significantly increased melanin area compared to the control group (Fig. 2, *B*–*D*). Therefore, this study concludes that MCH is a major factor in regulating melanin degradation, and changes in light levels regulate melanin degradation by affecting the secretion of MCH.

### Sleep pressure regulates the level of melanin degradation

**Figure 3:**
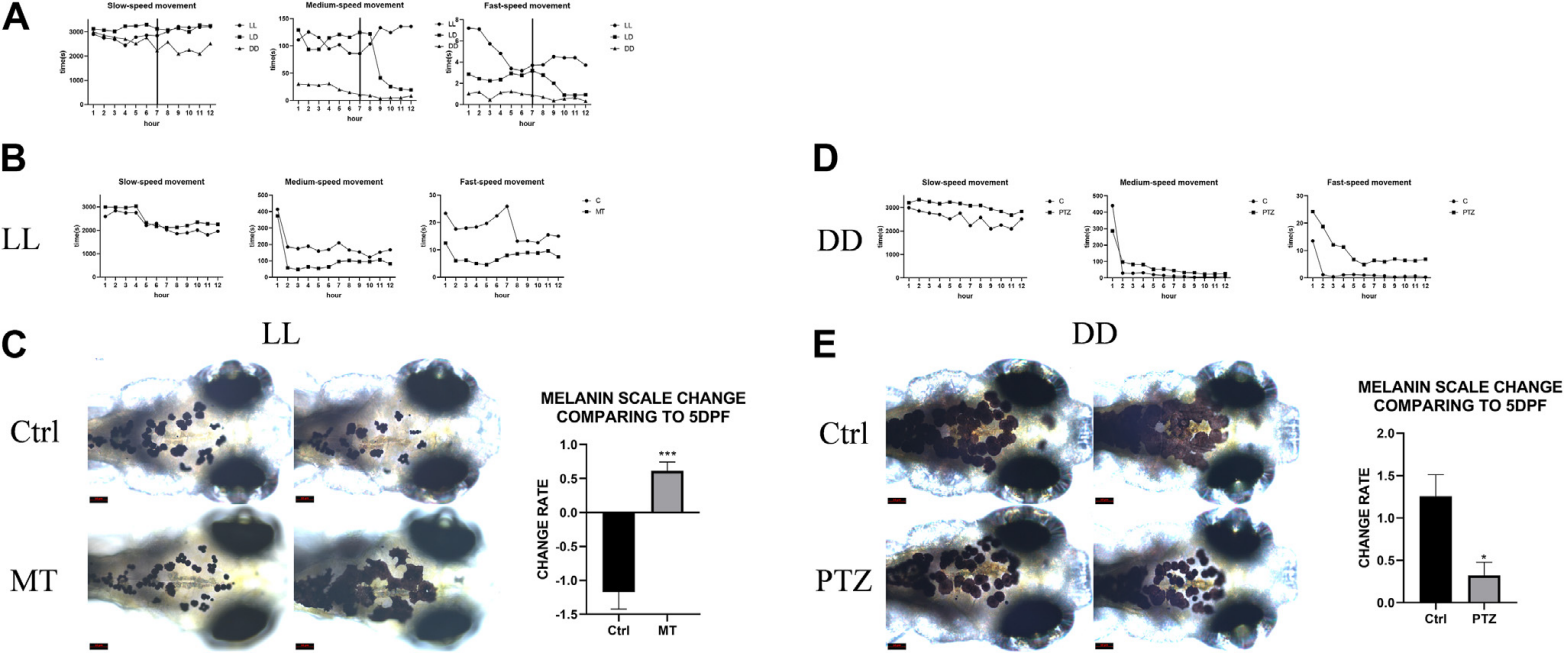
**Light patterns alter sleep pressure, thereby affecting the degradation of melanin**. *A*, records of slow, medium, and fast speed movements for 12 h at ZT7 under LL, LD, and DD conditions. *B*, records of slow, medium, and fast speed movements for 12 h starting at ZT7 in Ctrl and MT groups under LL condition. *C*, scale changes in surface melanin area of MT group and Ctrl group under LL and PTZ group and Ctrl group under DD at 7 dpf compared to 5 dpf. *D*, records of slow, medium, and fast speed movements for 12 h starting at ZT7 in Ctrl and PTZ groups under DD condition. *E*, scale changes in surface melanin area of PTZ group and Ctrl group under LL and PTZ group and Ctrl group under DD at 7 dpf compared to 5 dpf. Data are expressed as mean ± SEM (n = 6). ∗*p* < 0.05, ∗∗∗*p* < 0.001, *versus* Ctrl. dpf, days postfertilization; LD, light-dark cycle; MT, melatonin; PTZ, Pentetrazol; ZT, Zeitgeber time.

Current research extensively explores the relationship between MCH and sleep; however, no study has yet elucidated the connection between sleep and melanin. This study initially monitored the movement of embryos under LL, LD, and DD conditions using a behavioral tracking system. The results revealed no significant differences in initial slow speed movement times among the groups, but a notable decrease in slow speed movement time for the DD group in later stages. For medium speed movement time, LL was significantly higher than DD, while LD approached LL during the light phase and rapidly declined during the dark phase, nearing DD levels. In terms of fast speed movement, LL showed higher activity compared to DD, and LD exhibited a trend similar to LL during the late light phase, with a decline in activity during the dark phase, aligning with DD patterns (Fig. 3*A*). Moreover, by detecting the expression levels of fosab and hcrt under LL and DD conditions, we found that, compared to LL, fosab expression was significantly reduced, while hcrt expression was significantly increased under DD. This indicates that the LL condition indeed poses a sleep disturbance to the embryos (Fig. S1*C*). Thus, from the perspective of sleep pressure, it can be preliminarily concluded that the well-rested DD group, with low sleep pressure, showed low melanin degradation, whereas the LL group, which failed to sleep normally and had high sleep pressure, exhibited high melanin degradation.

Subsequently, zebrafish embryos were randomly divided into control and melatonin (MT) groups under LL conditions, and control and Pentetrazol (PTZ) groups under DD conditions to monitor sleep states and changes in melanin area. MT, a classic sleep-promoting drug, and PTZ, a common sleep-disrupting and convulsant drug, were used. Results indicated that under LL conditions, the MT group showed slightly lower slow speed movement time compared to the control, with both medium and fast speed movement times significantly lower (Fig. 3*B*). The melanin spot size in the MT group at 7 dpf significantly increased compared to 5 dpf (Fig. 3*C*). Under DD conditions, the PTZ group exhibited higher slow, medium, and fast movement times than the control (Fig. 3*D*), with a slight increase in melanin area at 7 dpf compared to 5 dpf, but significantly lower than the control group's increase (Fig. 3*E*).

We also conducted parallel comparisons using caffeine and found that the melanin changes and sleep behavioral data were consistent with the trends observed in the PTZ group (Fig. S1, *D*–*E*). Therefore, we conclude that sleep pressure is one of the factors affecting melanin degradation, and light conditions influence sleep pressure, thereby affecting melanin degradation.

### MCH and sleep pressure regulate each other in a cyclic manner, affecting the level of melanin degradation

**Figure 4:**
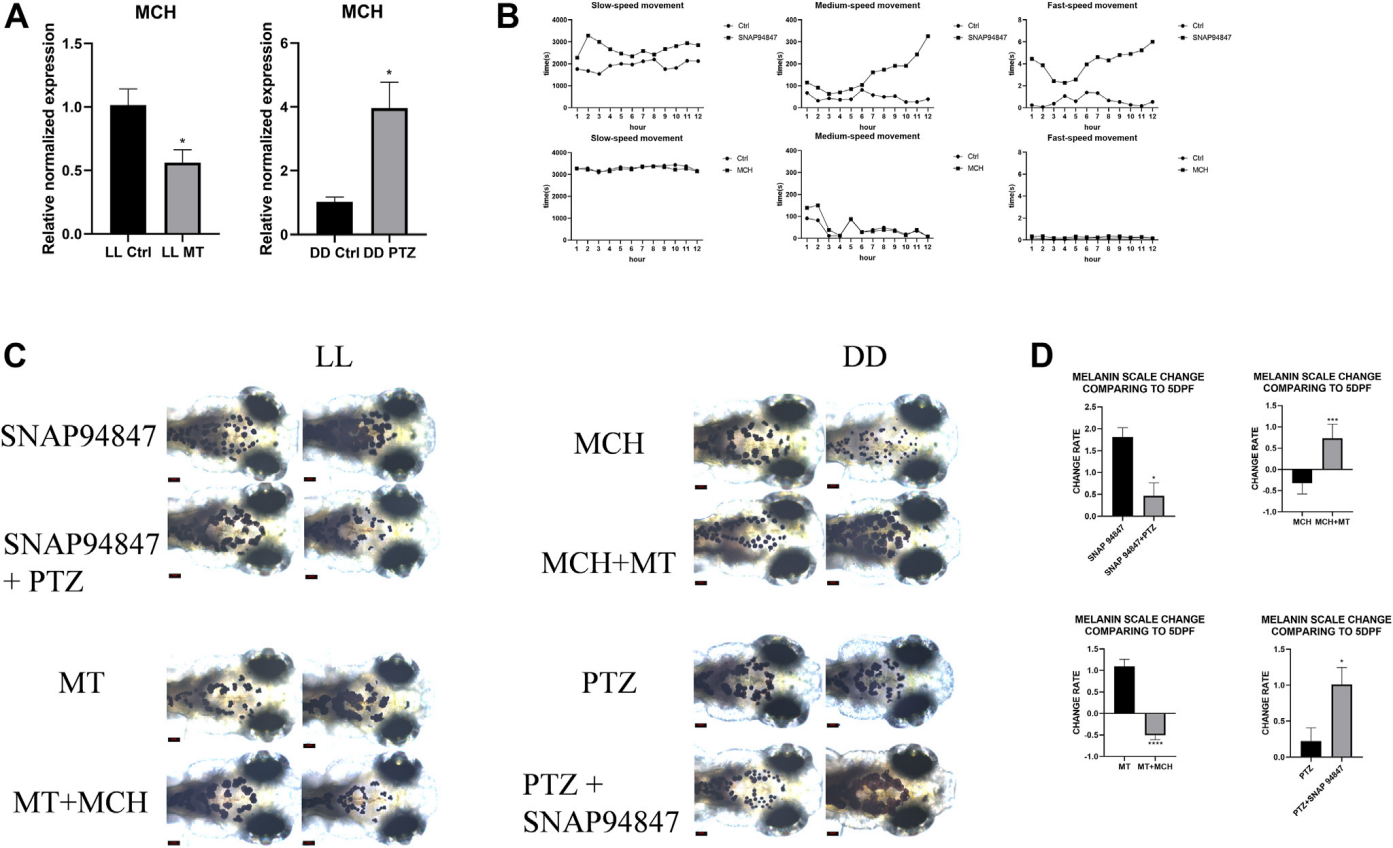
**MCH and sleep pressure form a cyclic regulatory loop affecting the degradation of melanin**. *A*, relative normalized expression of pmch in MT group and Ctrl group under LL and PTZ group and Ctrl group under DD. *B*, records of slow, medium, and fast speed movements for 12 h at ZT7 in SNAP94847 group and Ctrl group under LL condition, and MCH group and Ctrl group under DD condition. *C*, surface melanin of SNAP94847 group and SNAP94847+PTZ group, MT group and MT + MCH group, and MCH group and MCH + MT group, PTZ group and PTZ + SNAP94847 group at 5 dpf and 7 dpf, *D*, scale changes in surface melanin area of SNAP94847 group and SNAP94847+PTZ group, MT group and MT + MCH group, and MCH group and MCH + MT group, PTZ group and PTZ + SNAP94847 group at 7 dpf compared to 5 dpf. Data are expressed as mean ± SEM (n = 3). ns = no significance, ∗*p* < 0.05, ∗∗*p* < 0.01, ∗∗∗*p* < 0.001, ∗∗∗∗*p* < 0.0001, *versus* LL Ctrl, DD Ctrl, SNAP94847, MCH, MT, or PTZ. dpf, days postfertilization; MCH, melanin-concentrating hormone; MT, melatonin; PTZ, Pentetrazol; ZT, Zeitgeber time.

To explore the potential negative feedback regulation between MCH and sleep pressure in melanin degradation, we conducted RT-qPCR experiments to detect the pmch expression in embryos under LL (Ctrl and MT group) and DD (Ctrl and PTZ group) conditions. Additionally, we monitored the sleep states of embryos under LL (control and SNAP94847 group) and DD (Ctrl and MCH group) using a behavioral tracking system. The results showed that pmch expression was significantly reduced in the MT group and significantly increased in the PTZ group compared to the control group (Fig. 4*A*). The SNAP94847 group showed increased slow, medium, and fast speed movement times, while the MCH group did not show significant differences, consistent with existing research that MCH promotes sleep, and thus the DD group did not differ significantly from the control group. These findings suggest that MCH and sleep pressure can regulate each other in the mechanism of melanin degradation (Fig. 4*B*).

Subsequently, we randomly assigned embryos under LL to SNAP94847, SNAP94847+PTZ groups, MT and MT + MCH groups, and under DD to MCH, MCH + MT, PTZ and PTZ + SNAP94847 groups. We monitored melanin spots in embryos from 5 to 7 dpf to verify the mutual regulation between MCH and sleep pressure affecting melanin degradation. The results showed that at 7 dpf compared to 5 dpf, under LL conditions, the SNAP94847+PTZ group showed a significantly reduced trend in melanin area enlargement compared to the SNAP94847 group, and the MT + MCH group had a significantly smaller melanin area than the MT group. Under DD conditions, the MT + MCH group had a significantly larger melanin area than the MCH group, and the SNAP94847+PTZ group showed a more significant increase in melanin area enlargement than the PTZ group (Fig. 4, *C* and *D*).

### The MCH-sleep pressure cycle regulates the degradation of melanin through lysosomal hydrolysis and autophagic degradation

**Figure 5:**
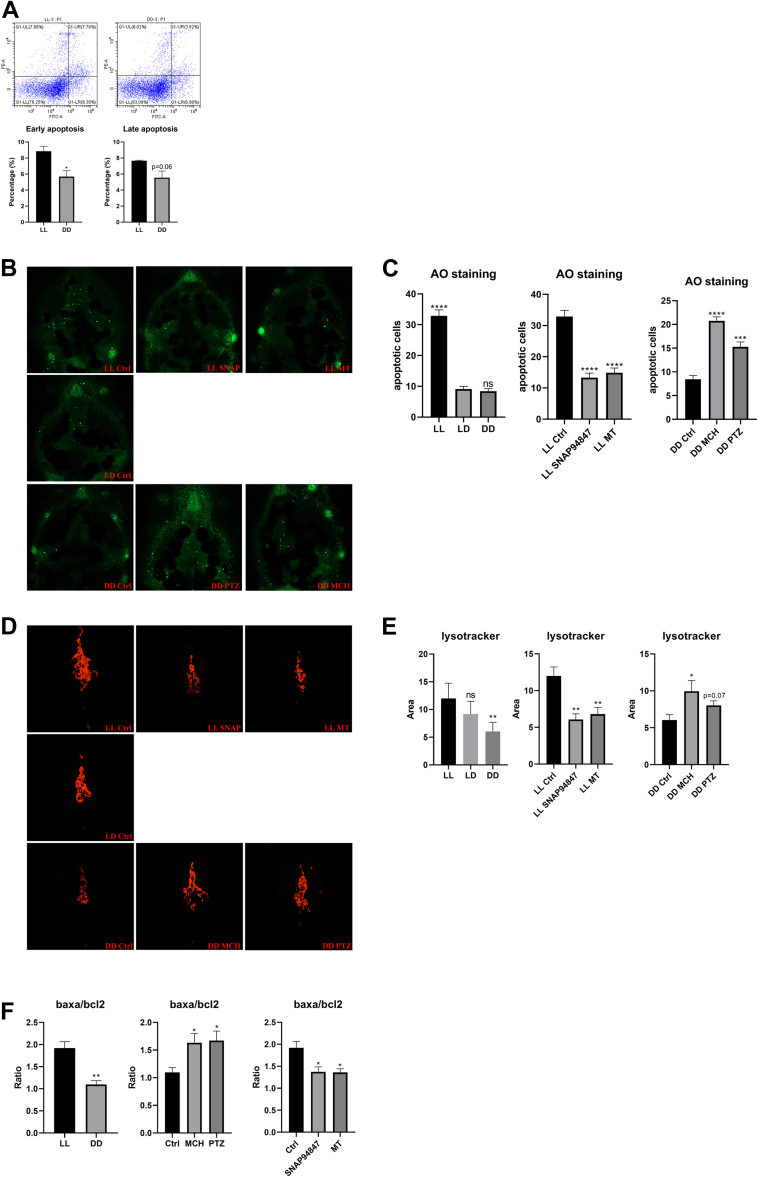
**The MCH-sleep pressure cycle regulates the degradation of melanin through autophagy and lysosomal degradation**. *A*, comparison of early and late apoptosis in zebrafish embryos by flow cytometry in LL and DD groups. *B*, AO staining images. *C*, statistics of apoptotic cells by AO staining. *D*, lysosomal staining images with LysoTracker. *E*, statistics of active lysosome area. *F*, ratio of baxa to bcl2 gene expression. The UL quadrant is (Annexin V-/PI+), which may be cell debris without a cell membrane or dead cells due to other reasons; the lower *left* quadrant represents normal (live) cells (Annexin V-/PI-); the UR quadrant is for late apoptotic cells (Annexin V+/PI+); the LR quadrant is for early apoptotic cells (Annexin V+/PI-). AO, acridine *orange*; DD, constant darkness; LL, constant light; LR, lower *right*; MCH, melanin-concentrating hormone; UL, upper *left*; UR, upper *right*.

Our preliminary experimental results indicate that there is a mutual regulatory relationship between MCH and sleep pressure, which in turn regulates the level of melanin degradation. Substances within cells are primarily degraded through two pathways: lysosomal hydrolysis and autophagic degradation. Studies have found that the degradation of melanosomes is also closely linked to these two pathways (15, 16). To explore the specific degradation mechanism of melanin, we first conducted flow cytometry on embryos under LL and DD conditions to assess the level of apoptosis. The results showed that, compared with the DD group, the early apoptosis in the LL group significantly increased, while late apoptosis was slightly higher but not statistically significant (*p* = 0.06), suggesting that the MCH-sleep pressure cycle may affect melanin degradation through apoptosis (Fig. 5*A*).

Subsequently, we used acridine orange (AO) staining and LysoTracker Red staining to detect the level of apoptosis and lysosomal activity in embryos under LL, LD, and DD conditions. The results showed that the LL group had the most apoptotic debris, while the LD and DD groups were significantly lower than the LL group, with no significant difference between LD and DD. The lysosomal activity in the LL group was more active than in the DD group, with no significant difference between LD and LL. Under LL conditions, the SNAP94847 and MT groups had significantly lower levels of apoptotic debris and lysosomal activity compared to the control group; under DD conditions, the MCH peptide and PTZ groups had significantly higher levels of apoptotic debris and lysosomal activity than the control group. These results further confirmed that the MCH-sleep pressure cycle regulates melanin degradation through apoptosis and lysosomal hydrolysis (Fig. 5, *B*–*E*).

Furthermore, we conducted RT-qPCR molecular experiments on BCL family related genes, baxa and bcl2, to assess the promotion and inhibition of autophagy (17). The baxa/bcl2 ratio in the LL group was significantly higher than in the DD group, while the DD group was close to 1, indicating that autophagy in the LL was significantly higher than in the DD, where autophagy was not apparent. Under LL conditions, the baxa/bcl2 ratios in the MT and SNAP94847 groups were lower than in the control group but still close to 1.5, indicating that although autophagy was inhibited, it still promoted autophagy overall; under DD conditions, the baxa/bcl2 ratios in the MCH peptide and PTZ groups were significantly higher than in the control group. These results are consistent with the previous staining results, further validating that the MCH-sleep pressure cycle regulates melanin degradation through autophagy (Fig. 5*F*).

Additionally, we detected the expression of the autophagy-related gene p62. Research indicates that p62 is a key factor in maintaining autophagic homeostasis. Under stress conditions, the expression of p62 can inhibit autophagy to protect cells, whereas suppressed p62 expression suggests that autophagy is occurring (18). The results showed that the p62 expression in the LD group was significantly higher than in the LL and DD groups, with the LL group significantly higher than the DD group. Under LL conditions, the p62 expression in the SNAP94847 and MT groups was lower than in the control group; under DD conditions, the p62 expression in the MCH group was significantly lower than in the control group, while the PTZ group showed no significant difference from the control group (Fig. S1*F*). The differences in p62 expression under LL, LD, and DD conditions in the control group suggest that p62 expression is low in DD due to the absence of external stressors. In contrast, LL and LD conditions involve light stimulation, with LD being a more stable environment. Thus, p62 is upregulated in LD to inhibit autophagy and protect cells, while it is silenced in LL, leading to increased autophagy. Under LL conditions, the SNAP94847 and MT groups showed reduced p62 expression and decreased autophagy due to the drugs mitigating environmental interference, shifting toward the stable state observed in DD. Conversely, in the DD background, the MCH and PTZ groups disrupted the stable state, leading to decreased p62 expression and increased autophagy.

In addition to the above, we explored the relationship between autophagy and lysosomal degradation using the classic autophagy drug hydroxychloroquine (HCQ). The results showed that, compared with the Ctrl group, the level of melanin scale change and the number of apoptotic fragments by AO staining was significantly reduced in the HCQ group, while there was no significant difference in lysosomal activity. We preliminarily concluded that autophagy and lysosomal degradation act as independent processes in melanin degradation (Fig. S1, *G*–*J*).

### MCH and PTZ promote the melanin degradation in the HaCat cell model

Previously, using the zebrafish model, we confirmed that the MCH-sleep pressure cycle can regulate lysosomal hydrolysis and autophagy levels, thereby affecting the melanin degradation. To explore the impact of MCH rather than other environmental factors or drugs on melanin degradation, this study employed the HaCat cell model to artificially transport melanin into HaCat cells (Fig. S2*A*). HaCat cells are peripheral tissue cells, distant from the central nervous system where MCH is secreted, and they do not produce melanin autonomously, while also exhibiting rhythmic expression closely related to sleep pressure. Initially, we cultured melanin-containing HaCat cells under LL and DD conditions with complete medium, and the results showed no change in melanin content under LL and DD conditions, indicating that light exposure does not directly promote melanin degradation (Fig. S2*B*).

Subsequently, we determined the safe concentrations of MCH, MT, PTZ, and SNAP94847 using the Cell Counting Kit-8 (CCK-8) assay. The results showed no significant differences between the MCH group and the control group at all concentrations, with white precipitation occurring at and above 2 mM, leading us to select 1 mM as the experimental concentration. For the MT group, no inhibition of cell activity was observed below 0.03 mM, thus 0.03 mM was chosen as the experimental concentration. The PTZ group showed stable cell viability starting at 2.5 mM, which was therefore selected as the experimental concentration. SNAP94847 exhibited a significant increase in cell viability between 10 μM and 2.5 μM, so 2.5 μM was chosen as the experimental concentration (Fig. S2*C*). We cultured melanin-containing HaCat cells with complete medium containing SNAP94847, PTZ, MCH peptide, and MT, as well as with blank complete medium. The results revealed that, compared to the control group, the melanin content in cells treated with SNAP94847 and MT showed no significant difference, while the melanin content in cells treated with PTZ and MCH peptide was significantly reduced. This result indicates that MT and SNAP94847 are not involved in the melanin degradation process, while also confirming the role of MCH and PTZ in promoting melanin degradation (Fig. S2*D*).

Furthermore, we detected the expression levels of the autophagy-related gene LC3 in the aforementioned groups using RT-qPCR. LC3 is a classic marker of autophagy, and its expression levels typically increase when autophagy is induced (19, 20). The results showed that, compared to the control group, the LC3 expression levels in the SNAP94847 and MT treatment groups showed no significant difference, while the LC3 expression levels in the MCH peptide and PTZ treatment groups were significantly increased (Fig. S2*E*), consistent with the trends observed in the zebrafish model, suggesting that autophagic degradation is the mode by which the MCH-sleep pressure cycle regulates melanin degradation.

## Discussion

To date, numerous studies have explored the relationship between MCH and sleep pressure, and it is widely believed that MCH is a sleep-promoting hormone with an antagonistic effect on growth hormone, regulating sleep-wake states (21, 22). The interaction between MCH and melanin is best known promoting melanin aggregation in vertebrate bony fish, where it is antagonistic to α-MSH. Under light backgrounds, upregulated MCH promotes melanin aggregation; when entering dark environments, MCH secretion is inhibited by upregulated α-MSH, leading to melanin dispersion (23). However, no study has reported how MCH specifically interacts with melanin. The preliminary experiments of this study verified the correlation between melanin aggregation under long-term light exposure and MCH through skin melanin area and RT-qPCR. Additionally, by controlling a single variable, the experimental condition's culture was replaced with buffer containing the commonly used melanin synthesis inhibitor PTU. Surprisingly, it was found that MCH’s role in promoting melanin aggregation seems unrelated to melanin production. Through the analysis of melanin content in zebrafish embryos, we found that the melanin aggregation under prolonged light exposure is due to reduced melanin content. Moreover, studies have shown that light exposure can induce oxidative stress through the generation of reactive oxygen species, which may influence cellular processes, including melanin degradation (24), but our study showed this reduction in melanin content is not significantly correlated with oxidative stress. These findings lead us to conclude that MCH is closely related to melanin degradation. Furthermore, the experimental results confirmed that zebrafish embryos secrete MCH to promote melanin aggregation under LL conditions, and it was also found that the expression level of MCH under LL conditions is significantly higher than under LD conditions, and the melanin content is significantly lower than under LD conditions. Therefore, we believe that MCH achieves melanin aggregation by reducing melanin content.

Soon after its initial discovery in salmon, MCH was rapidly identified in the brains of mammals. Currently, research on MCH in mammals primarily focuses on behaviors related to sleep, feeding, and energy balance (25). Studies on the relationship between MCH and the sleep-wake cycle have been extensively conducted, revealing that MCH accumulates during wakefulness, increasing sleep pressure, and is released upon falling asleep to maintain sleep homeostasis. After waking, MCH accumulates again, forming a cycle (26). However, there are virtually no studies reporting on the interplay between sleep pressure and melanin or the mutual regulation of MCH and sleep pressure on melanin. Given the three lighting conditions in this study, we preliminarily believe that the degradation of melanin may be related to sleep pressure. Utilizing sleep behavioral tracking and melanin scale analysis on embryos, we confirmed that sleep pressure can regulate the level of melanin degradation, promoting it when sleep pressure is high and inhibiting it when sleep pressure is low. Furthermore, by comparing the effects of cotreatment with MCH drugs and sleep pressure drugs to their individual treatments on surface melanin, we confirmed the existence of a cyclic feedback mechanism between MCH and sleep pressure that jointly regulates the level of melanin degradation.

**Figure 6:**
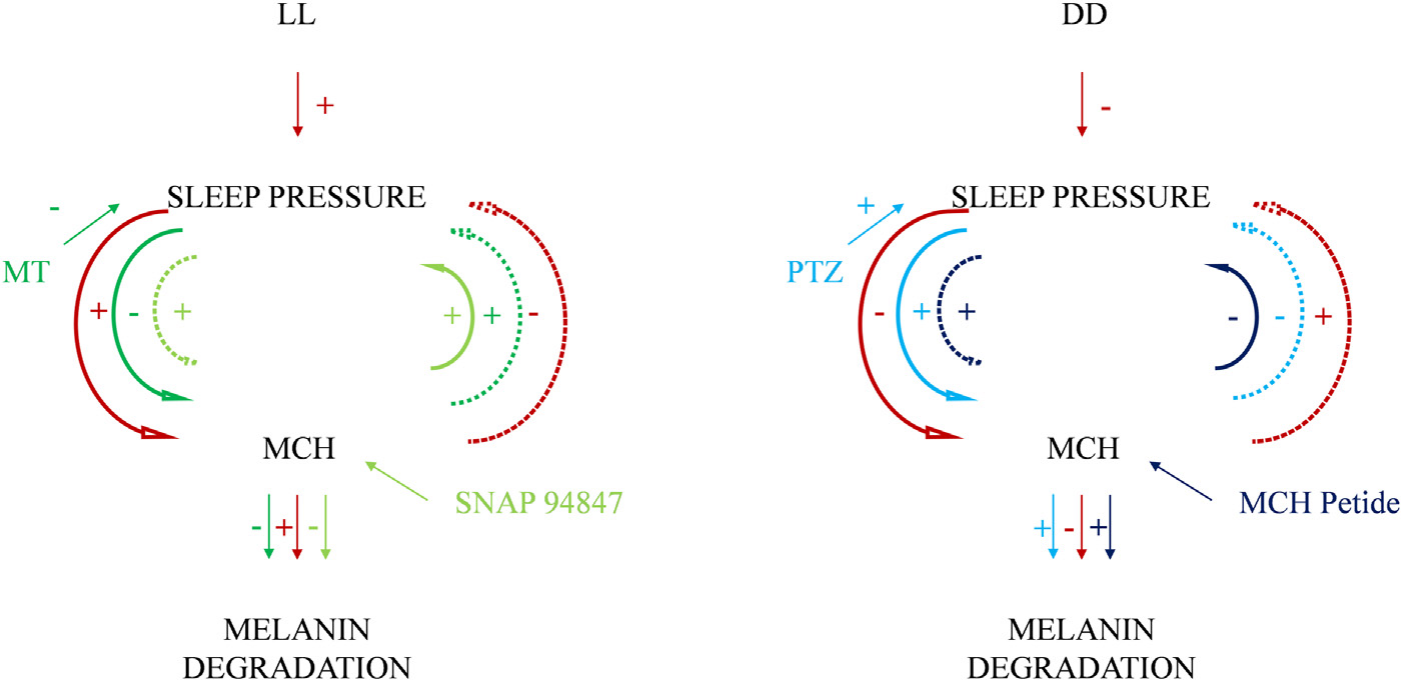
**MCH-sleep pressure loop**. MCH, melanin-concentrating hormone.

Under LL conditions, light keeps embryos awake, increasing sleep pressure and MCH secretion, leading to increased melanin degradation and promoting sleep. However, continuous light keeps embryos continuously awake, maintaining MCH expression and the increased level of melanin degradation. When MT is added, it induces sleep, reduces sleep pressure, and MCH neurons become inactive after the onset of sleep, leading to decreased MCH expression, a decrease in the level of melanin degradation, and gradual awakening. However, an excess of MT keeps embryos in a sleep state, maintaining a reduced level of melanin degradation. When the MCH-receptor inhibitor (SNAP94847) is added, MCH cannot bind to its receptor, making embryos more active than the control group, increasing sleep pressure and promoting MCH secretion to induce sleep. However, due to the binding of MCH-receptor inhibitor with MCH receptors, the secreted MCH cannot bind to the receptors, and negative feedback reduces the expression of MCH genes, keeping embryos continuously awake and reducing the level of melanin degradation. Under DD conditions, darkness makes embryos tend to sleep, reducing sleep pressure and MCH secretion, leading to a decrease in the level of melanin degradation and a tendency toward wakefulness. However, continuous darkness keeps embryos in an inactive state, maintaining low MCH expression and a reduced level of melanin degradation. When PTZ is added, it induces wakefulness, increases sleep pressure, activates MCH neurons, leading to increased MCH expression, an increase in the level of melanin degradation, and a gradual tendency toward sleep. However, an excess of PTZ keeps embryos in a state of wakefulness, maintaining an increased level of melanin degradation. When MCH is added, after being taken up by the embryos, it maintains a steady state of sleep, reduces sleep pressure, and tends to inhibit MCH secretion to induce sleep. However, due to the excessive binding of MCH with receptors, embryos continue to remain in a sleep state and continuously increase the level of melanin degradation (Fig. 6).

In this study, we employed flow cytometry, LysoTracker Red staining, AO staining, and RT-qPCR analysis of relevant genes to reveal that melanin degradation is regulated through autophagy and lysosomal degradation pathways. We also used the classic autophagy inhibitor HCQ to preliminarily determine that autophagy and lysosomal degradation act as independent processes in melanin degradation (27). Furthermore, we utilized the HaCat cell model to independently verify whether the degradation of melanin transported to keratinocytes is regulated by drugs such as MCH and the modes of its degradation. Since HaCat cells do not secrete MCH, do not produce melanin, and serve as a reservoir for melanin after its generation and transport (28), and are normally regulated by circadian genes (29), we conducted research by artificially transporting melanin into the cells. Using melanin content analysis and RT-qPCR, our results confirmed that MCH induces melanin degradation through the autophagy pathway, while SNAP94847 and MT did not cause a reduction in melanin or changes in cellular autophagy levels. Notably, PTZ was found to reduce melanin and increase autophagy levels, which may be related to circadian rhythms, as sleep-wake states have always been considered an important parameter in rhythm research (30). Therefore, the relationship between circadian rhythms and melanin should be given attention in subsequent studies.

Although zebrafish share many conserved pathways with mammals, there are significant differences in the neural and hormonal mechanisms underlying sleep and pigmentation regulation between fish and humans. For instance, the sleep regulatory network in mammals involves complex interactions among the suprachiasmatic nucleus, the ventrolateral preoptic area, and other brain regions, which are not fully recapitulated in zebrafish (31). Despite these differences, our findings in zebrafish provide a simplified yet powerful model for dissecting the fundamental mechanisms linking sleep pressure and melanin regulation. While these results cannot be directly extrapolated to humans, they offer important insights and a foundation for future studies in more complex systems.

Our current study primarily focuses on establishing the correlation between sleep pressure, MCH expression, and melanin degradation. Recent studies have demonstrated that sleep pressure, often mediated by factors such as adenosine accumulation, can influence gene expression and synaptic plasticity (32). These findings suggest that sleep pressure may modulate MCH expression through similar mechanisms, although the specific pathways remain to be fully characterized. We recognize the need for further investigation to elucidate the direct or indirect nature of these interactions. Future work from our group will aim to dissect the molecular mechanisms by which MCH influences autophagy and lysosomal activity. We plan to explore potential signaling cascades, such as the involvement of adenosine receptors or other neuroendocrine pathways, which have been implicated in both sleep regulation and melanin dynamics.

## Experimental procedures

Exogenous MCH and HCQ were purchased from China MCE. SNAP94847 was purchased from Selleck. PTZ, MT, and NaOH were purchased from Aladdin. PBS was purchased from Solarbio. Fetal bovine serum (FBS) was purchased from Corning. DCFH-DA was purchased from Yuanye. Caffeine was kindly provided by Sun-Hi Biotech.

Total RNA Column Extraction Kit was purchased from Vazyme. HiScript III RT SuperMix for quantitative PCR (qPCR) (+gDNA wiper) Reverse Transcription Kit was purchased from Vazyme. ChamQ Universal SYBR qPCR Master Mix was purchased from Vazyme. VeZol Reagent was purchased from Vazyme.

Annexin V-FITC/propidium iodide (PI) Apoptosis Kit was purchased from Multi Sciences, and AO fluorescent staining kit was purchased from Yuanye Bio-Technology LysoTracker Red was purchased from Solarbio.

### Animals

WT/AB strain zebrafish (*Danio rerio*) were purchased from the China Zebrafish Resource Center (Wuhan, China). Adult zebrafish were housed in a breeding system with a 14-h light and 10-h dark cycle, temperature ranges of 26.0 to 29.0 °C, water conductivity of 500 to 550 μS/cm, and pH of 7.0 to 8.0. Zebrafish were fed with live brine shrimp larvae, twice daily. On the day before the experiment, after the zebrafish had been fed for 1 h, the healthy ones were selected and placed in zebrafish breeding tanks. The males and females were separated by a partition at a ratio of 1:1. The following morning, the partition was removed, allowing the zebrafish were free to mate. Then, the fertilized eggs at the bottom of the tank were collected after 1 h. Healthy fertilized eggs were selected and placed in an incubator at 28.5 ± 1 °C. Considering that the nervous and circadian systems of zebrafish embryos are essentially mature by 5 dpf, in this experiment, embryos were transferred from the incubator to the fish room at 3 dpf cultured under a temperature of 28.5 ± 1 °C and a 14:10 LD cycle to establish a model simulating the normal circadian rhythm of zebrafish.

All zebrafish experiments complied with the Organization for Economic Co-operation and Development Test Guidelines (TG203 and TG236). All animal experimental procedures were conducted in accordance with the ethical standards of Guangdong University of Technology.

### Cell culture and reagent

Human immortalized epidermal cells (HaCat) and complete HaCat culture medium were purchased from Shanghai Zhongqiao Xinzhou Biotechnology Co., Ltd. The CCK-8 was provided by TransGen Biotech. HaCat cells were cultured in a humidified incubator with 5% CO_2_ at 37 °C.

### Zebrafish embryo collection and drug treatment

Zebrafish embryos were prepared according to previously described methods. Briefly, paired matings (aged: 6–12 months) were used to generate zebrafish embryos. Embryos were selected for further experiments based on microscopic examination. The embryos were then placed in Holt-Buffer and incubated in an incubator at 28.5 ± 1 °C until 3 dpf. The status of the embryos was checked every 24 h, and any poorly developed or dead embryos were removed. At 3 dpf, the embryos were transferred from the incubator to a zebrafish facility with a controlled LD cycle and constant temperature, ensuring normal light exposure for all embryos, and cultured until 5 dpf. The 5 dpf embryos were arranged on 24-well plates (8 embryos/well) or 6-well plates (25 embryos/well).

To investigate whether light influences melanin scale change through the regulation of MCH, this study randomly assigned zebrafish embryos at 5 dpf to LL, LD, and DD conditions and treated them with Holt-Buffer until 9 dpf. During the experiment, the surface melanin area of the embryos was recorded on 5, 7, and 9 dpf.

To determine whether the differences in melanin scale change on the surface of zebrafish are related to melanin production or degradation, this study employed the classic melanin synthesis inhibitor PTU. After assigning the 5 dpf embryos to LL, LD, and DD environments, they were cultured with Holt-Buffer containing 0.02 mM PTU until 9 dpf. The culture medium was replaced every 48 h, and the surface melanin area and content of the embryos were recorded on 5, 7, and 9 dpf.

To explore whether the differences in melanin pigmentation on the surface of embryos under LL, LD, and DD conditions are caused by MCH or sleep pressure, this study treated 5dpf embryos with Holt-Buffer until 7dpf, and then used RT-qPCR to detect the expression levels of MCH-related gene pmch and sleep-related genes fosab and hcrt. The embryos under LL and DD conditions were treated with the 2.5 μM DCFH-DA probe to detect the levels of oxidative stress. Concurrently, a 12-h track behavior experiment was conducted on 5 dpf embryos at ZT7 (Zeitgeber time) to assess sleep patterns under LL, LD, and DD conditions.

To verify the correlation between melanin differences on the surface of zebrafish under LL, LD, and DD environments and MCH, the study initially treated 5 dpf embryos with Holt-Buffer until 7 dpf and detected the expression levels of the MCH-related gene pmch using RT-qPCR. Subsequently, 5 dpf embryos were randomly assigned to LL and DD conditions, with embryos in the LL environment divided into a control group and a SNAP94847 group, and those in the DD environment divided into a control group and an MCH group. The control group embryos were treated with Holt-Buffer, the SNAP94847 group embryos with Holt-Buffer containing 0.002 mM SNAP94847, and the MCH group embryos with Holt-Buffer containing 1 mM MCH peptide. All groups were treated until 7 dpf, and the surface melanin area was recorded on 5 and 7 dpf.

To verify the correlation between the differences in surface melanin of zebrafish under LL, LD, and DD conditions and sleep pressure, this study initially conducted a 12-h track behavior experiment on embryos at 5 dpf at ZT7. Subsequently, 5 dpf embryos were randomly assigned to LL and DD environments. In the LL environment, embryos were divided into a control group and an MT group; in the DD environment, embryos were divided into a control group and a PTZ group. The control group embryos were treated with Holt-Buffer, the PTZ group embryos with Holt-Buffer containing 1.5 mM PTZ, and the MT group embryos with Holt-Buffer containing 0.8 mM MT. All groups were treated until 7 dpf, and the surface melanin area and track behavior data of the embryos in each group were recorded.

To verify the correlation between melanin degradation and sleep disruption, embryos were randomly divided into a Ctrl group and a Caff group, and treated under LL conditions from 5 to 7 dpf, and the surface melanin area and track behavior data of the embryos in each group were recorded. The Caff group was treated with Holt-Buffer containing 10 mM Caffeine.

To explore the potential feedback regulatory mechanisms between MCH and sleep pressure, this study randomly assigned zebrafish embryos at 5 dpf to LL and DD environments. In the LL environment, embryos were divided into MT, MT + MCH, SNAP94847, and SNAP94847+PTZ groups; in the DD environment, embryos were divided into PTZ, PTZ + SNAP94847, MCH, and MCH + MT groups. The MT group embryos were treated with Holt-Buffer containing 0.8 mM MT, the PTZ group embryos with Holt-Buffer containing 1.5 mM PTZ, the SNAP94847 group embryos with Holt-Buffer containing 0.002 mM SNAP94847, the MCH group embryos with Holt-Buffer containing 1 mM MCH, the MCH + MT group embryos with Holt-Buffer containing 1 mM MCH and 0.8 mM MT, and the SNAP94847+PTZ group embryos with Holt-Buffer containing 0.002 mM SNAP94847 and 1.5 mM PTZ. All groups were treated until 7 dpf, and the surface melanin area of the embryos was recorded. Simultaneously, the expression levels of the MCH-related gene pmch were detected by RT-qPCR in the control group and MT group under the LL environment, as well as in the control group and PTZ group under the DD environment. In addition, the track behavior data of the embryos in the control group and SNAP94847 group under the LL environment and in the control group and MCH group under the DD environment were recorded.

To explore the relationship between autophagy and lysosomal degradation, embryos were randomly assigned to Ctrl and HCQ groups and treated under LL conditions from 5 dpf to 7 dpf, and the surface melanin area of the embryos in each group were recorded. The HCQ group was treated with Holt-Buffer containing 80 μM HCQ.

### Zebrafish surface melanin scale assay

Zebrafish embryos at 5, 7, and 9 dpf were imaged using the Zeiss Axio Imager.2 microscope imaging system to document melanin distribution from a top-view perspective, with eight embryos per group. The melanin from the head to one-quarter of the yolk sac was quantified using ImageJ software (National Institutes of Health, USA). The region of interest was outlined as previously described to obtain the integrated density, and the average pixel values were compared among different treatment groups to determine the melanin area size.

### Zebrafish melanin content assay

Embryos at 5, 7, or 9 dpf were grouped into sets of 25, with three technical replicates per group, and transferred to 1.5 ml centrifuge tubes. The embryos were washed three times with PBS solution at pH 7.4 and then homogenized in the PBS solution using a grinding rod until no visible clumps remained. The samples were then centrifuged at 12,500 rpm for 5 min at 4 °C. After centrifugation, the supernatant was discarded, and the black pellet was retained. Subsequently, 100 μl of 1 M NaOH solution was added, and the samples were incubated at 85 °C for approximately 50 min, with occasional shaking to ensure complete dissolution of the melanin pellet in the NaOH solution. The dissolved melanin from each group was then transferred to a 96-well plate. The relative melanin content was determined by measuring the absorbance at 450 nm using a TriStar2S plate reader.

### Sleep behavioral assay

Zebrafish embryos at 5 dpf were placed individually in 48-well plates and subjected to behavioral tracking under different lighting conditions to assess sleep pressure. Specifically, embryos were exposed to 12 h of light for the LL group, a 7-h light: 5-h dark cycle for the LD group, and 12 h of darkness for the DD group. Movement was monitored to evaluate slow, medium, and fast speed movements, thereby assessing sleep pressure.

To verify the effects of MT and PTZ on sleep, 5 dpf embryos under LL conditions were randomly assigned to control and MT groups, while those under DD conditions were assigned to control and PTZ groups. The embryos were monitored for 12 h to assess slow, medium, and fast speed movements, thereby evaluating sleep pressure.

To investigate whether MCH affects sleep pressure, 5 dpf embryos under LL conditions were randomly assigned to control and SNAP94847 groups, and those under DD conditions were assigned to control and MCH peptide groups. The embryos were monitored for 12 h to assess slow, medium, and fast speed movements, thereby evaluating sleep pressure.

All experiments commenced at ZT7, with a duration of 12 h, corresponding to the proportion of the light and dark phases in the zebrafish circadian rhythm pattern.

### Flow CytoMetry assay

Embryos under LL, LD, and DD conditions were treated from 5 dpf to 7 dpf, then transferred to 2 ml centrifuge tubes and centrifuged at 5000 rpm for 3 min. After centrifugation, the embryos were washed twice with distilled water and placed on a 70 μm cell strainer for grinding. PBS (2 ml) was added to rinse the cells off the strainer, and the supernatant was removed by centrifugation. Subsequently, 400 μl of 0.25% trypsin + 100 μl of 1 mg/ml collagenase was added to resuspend the cells, and the mixture was incubated at 37 °C for 5 min to digest the tissue. Then, 500 μl of PBS containing 5% FBS was added to stop the digestion. Cells were imaged and counted using a Corning Cytosmart cell counter.

Following this, cells were collected by centrifugation, and 500 μl of 1 × working solution (prepared by diluting 5 × binding buffer with double-distilled water) was used to resuspend the cells. To each tube, 5 μl of Annexin V-FITC and 10 μl of PI were added, and the mixture was gently vortexed and incubated at room temperature in the dark for 5 min.

On the flow cytometer, the voltage for forward scatter, side scatter, and fluorescence channels was adjusted using a blank tube, and the compensation for the fluorescence channels was set using single-stain tubes. Annexin V FITC-A was detected through the FITC channel (Ex = 488 nm; Em = 530 nm), and PI was detected through the PI PE-A detection channel (Ex = 535 nm; Em = 615 nm).

### AO staining assay

To investigate the association between melanin degradation and cell apoptosis, we treated zebrafish embryos from various groups to 7 dpf, after which the treatment solution was replaced with Holt-Buffer containing 20 μg/ml AO staining solution and incubated for 30 min. Following the treatment, the embryos were washed three times with Holt-Buffer to remove excess staining solution. Subsequently, the distribution of apoptotic cell debris around the melanin layer of the embryo's head was recorded using the LSM 800 laser confocal microscope to assess the level of cell apoptosis.

### LysoTracker assay

To explore whether the observed melanin degradation is associated with lysosomal hydrolysis, zebrafish embryos from various groups were treated to 7 dpf. The treatment medium was then replaced with Holt-Buffer prewarmed to 28.5 °C containing 50 nM LysoTracker, and the embryos were incubated for 30 min. After the treatment, the embryos were washed three times with Holt-Buffer to remove excess dye. Subsequently, the LSM 800 laser confocal microscope was used to record the area of red fluorescence around the melanin layer of the embryo's head to assess the extent of lysosomal hydrolysis.

### Cytotoxicity assay

HaCat cells were plated into a 96-well plate at a density of 4 × 10^5^ cells per well, with 100 μl of HaCat complete medium added to each well, in five replicates. After a 24-h incubation, the medium was aspirated, and the cells were washed twice with PBS. Subsequently, 100 μl of solution was added to each well 24 h later. The experimental groups were treated with MCH, SNAP94847, PTZ, and MT, dissolved in HaCat complete medium to achieve various concentrations: SNAP94847 (10, 5, 2.5, 1.25, 0.625, 0.3125, 0.15625, 0.078125, and 0.0390625 μM), PTZ (10, 5, 2.5, 1.25, 0.625, 0.3125, 0.15625, 0.078125, and 0.390625 mM), MCH (4, 2, 1, 0.5, 0.25, 0.125, 0.0625, 0.03125, and 0.015625 mM), and MT (0.12, 0.06, 0.03, 0.015, 0.0075, 0.00375, 0.001875, 0.0009375, and 0.00046875 mM). The control group was cultured with HaCat complete medium only.

After another 24-h incubation, the cells were washed twice with PBS, and 100 μl of 10% CCK-8 reagent was added to each well for a 50-min reaction. Absorbance was measured at 450 nm. All experiments were conducted independently with five replicates.

### Detection of melanin degradation

To investigate whether light exposure directly regulates melanin degradation, HaCat cells were seeded at a density of 4 × 10^5^ cells per well in 6-well plates and cultured overnight in complete HaCat medium. The medium was then replaced with a low-serum medium containing 0.1% melanin (MCE) and 0.5% FBS (Zhong Qiao Xin Zhou) and cultured for 2 days. Subsequently, the medium was switched back to complete medium, and the cells were cultured under LL and DD conditions overnight. The medium was then replaced with 0.25% trypsin cell digestion solution (Zhong Qiao Xin Zhou) to detach the cells, which were transferred to 1.5 ml centrifuge tubes, centrifuged, and the supernatant was discarded. To the pellet, 100 μl of 1 M NaOH solution was added, and the tubes were incubated at 85 °C for approximately 40 min, with occasional shaking to ensure complete dissolution of the melanin pellet in the NaOH solution. The dissolved melanin from each group was then transferred to a 96-well plate, and the relative melanin content was determined by measuring the absorbance at 450 nm using a TriStar2S plate reader.

To explore whether MCH and PTZ directly promote melanin degradation and whether MT and SNAP94847 are involved in the melanin degradation process, HaCat cells were seeded at a density of 4 × 10^5^ cells per well in 6-well plates and cultured overnight in complete HaCat medium. The medium was then replaced with a low-serum medium containing 0.1% melanin (MCE) and 0.5% FBS (Zhongqiao Xinzhou) and cultured for 2 days. Subsequently, the medium was switched back to complete medium, and the cells were divided into groups treated with 1 mM MCH, 2.5 μM SNAP94847, 1 mM PTZ, and 0.03 mM MT overnight. The relative melanin content was then determined as described above.

### RNA isolation and complementary DNA preparation

Total RNA from embryos of each group was extracted using a Total RNA Column Extraction Kit when the embryos reached 7 dpf, following the manufacturer's protocol. The quality of the total RNA was assessed using an Agilent 2100 Bioanalyzer (Agilent), agarose gel electrophoresis, and a nanophotometer. The HiScript III RT SuperMix for qPCR (+gDNA wiper) (Vazyme #R323) includes a genomic DNA removal module to thoroughly eliminate residual genomic DNA in the RNA template. Additionally, Vazyme #R323 contains all the components required for the reverse transcription reaction, allowing the process to be completed by simply adding template RNA.

### Quantitative real-time polymerase chain reaction

RT-qPCR was performed using ChamQ Universal SYBR qPCR Master Mix (Vazyme). CFX96 Touch Real-Time PCR Detection System (Bio-Rad) was used to perform three PCR tests for each RNA sample. Each sample contained 0.5 μl complementary DNA and 10 μl 2X ChamQ Universal SYBR qPCR Master Mix. Relative gene expression levels were assessed using the 2^−ΔΔCt^ formula, and then β-actin gene expression was normalized to target gene expression. All primers were independently designed and validated (Table 1).

**Table 1:** The primers for target genes

Primer	Front primer sequence (5′-3′)	Rear primer sequence (3′-5′)
pmch	ATCATCGTGGTGGCTGACTC	CAGCATGGCCGATACACTCT
p62	GTAACCCCAACTCCTCCTGC	TCCTGTCGAAGGATCCACCT
lc3	GGAGAGAAGCAACTGCCGAT	CGTCTTCGTCTCTTTCCCGT
β-actin	GCCGTGACCTGACTGACTAC	GGGCACCTGAACCTCTCATT
GADPH	GGCACAGTCAAGGCTGAGAATG	ATGGTGGTGAAGACGCCAGTA
baxa	TACTTTGCCTGTCGCCTTGT	CAGCGAGGAAAACTCCGACT
bcl2	GCGCTTCAACGCAGTCATAG	GAGGGAAGGCGTGTGAAGAA
fosab	CGATACACTGCAAGCTGAAAC	CGGCGA GGATGAACTCTAAC
hcrt	ACTCTACGAGATGCTGTGCC	CTTGATTCCGCGAGTTGTGC

### Statistical analysis

All analyses were performed using GraphPad Prism (version 8.0, GraphPad Software, Santiago de Chile, United States). Differences between groups were analyzed using one-way analysis of variance (ANOVA). Differences were considered statistically significant when *p* values < 0.05.

## Data availability

The data that support the findings of this study are available on request from the corresponding author. The data are not publicly available due to privacy or ethical restrictions.

## Supporting information

This article contains supporting information.

## Declaration of generative AI and AI-assisted technologies

We used the generative artificial intelligence (GenAI) tool Kimi to prepare this manuscript and related supplementary documents. Kimi is an AI writing assistant used to improve writing quality, reduce text duplication rates, and add more human touch to the work. We utilized Kimi to assist in writing various sections of the paper, including the abstract, introduction, main text, and conclusion. The reason for using Kimi was to enhance the efficiency and quality of the paper writing.

## Ethical statement

All animal experiments were conducted in strict accordance with the National Institutes of Health (NIH) guide for the care and use of Laboratory animals (publication No. 85–23, revised in 1985). Adult zebrafish were monitored for signs of distress, such as lethargy, loss of appetite, or abnormal swimming behavior, and were euthanized if these signs were observed. The zebrafish were also euthanized at the end of the experiment to prevent any potential suffering. Embryos were monitored for signs of distress, such as abnormal swimming behavior or lack of movement, and were euthanized if these signs were observed. The embryos were also euthanized at the end of the experiment to prevent any potential suffering. The protocol was approved by the Institutional Animal Care and Use Committee (IACUC) of Guangdong Provincial People’s Hospital, and all efforts were made to minimize suffering. The ethics approval/permit number for the use of animals in this study is S2024 to 849-01.

## Conflict of interest

The authors declare that they have no conflicts of interest with the contents of this article.

## Figures and Tables

**Fig. S2:**