# Supplementary material for: Melanin concentrating hormone-sleep pressure loop regulates melanin degradation through both autophagic degradation and lysosomal hydrolysis in zebrafish
Source: J Biol Chem. 2025 Apr 8;301(5):108486. doi: 10.1016/j.jbc.2025.108486 (PMC12147225; doi:10.1016/j.jbc.2025.108486)
Supplement: Supporting Information [file mmc1.docx]

**Supporting figure legends**

Figure S1. (A) The embryos under LL and DD conditions were stained with the DCFH-DA probe at 7 dpf (n = 15), (B) Quantification analysis of ROS fluorescent areas in embryos under LL and DD conditions (n = 15), (C) Relative normalized expression of hcrt and fosab genes in embryos under LL and DD conditions at 7 dpf (n = 30), (D) Surface melanin and the scale changes of Ctrl and Caff groups at 5 dpf and 7 dpf under DD condition (n = 8), (E) Records of slow, medium, and fast speed movements for 12 hours starting at ZT7 in Ctrl and Caff groups under DD condition (n = 8), (F) Relative normalized expression of p62, (G) Surface melanin of Ctrl and HCQ groups at 5 dpf and 7 dpf under LL condition (n = 8), (H) Melanin scale changes of Ctrl and HCQ groups at 5 dpf and 7 dpf under LL condition (n = 8), (I) AO and Lysosomal staining images of Ctrl and HCQ groups at 7 dpf (n = 10), (J) Statistics of apoptotic cells and active lysosome area. (n = 10) Data are expressed as mean ± SEM. ns = no significance, *P < 0.01, **P < 0.01, *P < 0.01, ***P < 0.001, versus control.

Figure S2. The promotion of melanin degradation by PTZ and MCH has been validated in the HaCaT cells. (A) Melanin was artificially transported into HaCaT cells, (B) Cytotoxic testing of PTZ, MT, MCH, and SNAP94847, (C) Impact of light conditions on melanin content, (D) Impact of PTZ, MT, MCH, and SNAP94847 treatments on melanin content in HaCaT cells, (E) Relative normalized expression of LC3. *P < 0.05, **P < 0.01, ***P < 0.001, ****P < 0.0001, versus control.
